# A105 ENDOSCOPIC MUCOSAL RESECTION AND ENDOSCOPIC SUBMUCOSAL DISSECTION FOR ILEOCECAL VALVE NEOPLASIA: A SYSTEMATIC REVIEW AND META-ANALYSIS

**DOI:** 10.1093/jcag/gwab049.104

**Published:** 2022-02-21

**Authors:** K Donaldson, A A Arif, H Qian, E Lam, N C Shahidi

**Affiliations:** 1 University of British Columbia, Department of Medicine, Vancouver, BC, Canada; 2 Centre for Health Evaluation and Outcome Sciences, Vancouver, BC, Canada; 3 St. Paul’s Hospital, Division of Gastroenterology, Vancouver, BC, Canada

## Abstract

**Background:**

Neoplastic lesions at the ileocecal valve (ICV) represent a complex lesion subgroup given the unique anatomical characteristics of this location. Both endoscopic mucosal resection (EMR) and endoscopic submucosal dissection (ESD) are established techniques for colorectal neoplasia but comparative analyses for ICV lesions are lacking.

**Aims:**

Evaluate the performance of EMR and ESD for ICV neoplasia.

**Methods:**

Between Jan 2000 to Aug 2021, two authors independently searched MEDLINE, EMBASE and Cochrane Libraries for relevant citations evaluating the performance of either EMR and/or ESD for ICV neoplasia; defined as lesions involving at least one component of the ICV complex. The rate of technical success (complete removal of all neoplastic tissue during index procedure of those lesions deemed amenable to endoscopic resection), clinically significant post-endoscopic resection bleeding (CSPEB), delayed perforation, and recurrence were assessed. Meta-analysis was performed using a random-effects model.

**Results:**

Nine studies (367 patients, 252 EMR, 115 ESD) were included in the analysis. Successful removal of all visible neoplastic tissue of those deemed amenable to endoscopic resection was 98.1% (EMR 99.6%, ESD 97.4%). Of note, only 2 studies, both assessing EMR, provided data on lesions which were not considered for endoscopic resection ranging from 5.6–23.7%. Average procedure time ranged from 45–49 minutes for EMR and 52–191 minutes for ESD. Clinically significant post-endoscopic resection bleeding occurred in 6.2% (EMR 9.4%, ESD 4.4%). Delayed perforation occurred in 0.6% (EMR 0.4%, ESD 2.0%). Recurrence occurred in 3.1% (EMR 13.2%, ESD 1.9%).

**Conclusions:**

Endoscopic resection, both with EMR and ESD, demonstrates high technical success and good adverse event profiles amongst ICV neoplasia deemed amenable for endoscopic resection.

**Funding Agencies:**

None

